# Pan-Cancer Prognostic Role and Targeting Potential of the Estrogen-Progesterone Axis

**DOI:** 10.3389/fonc.2021.636365

**Published:** 2021-07-12

**Authors:** Yu-ting Shen, Xing Huang, Gang Zhang, Bo Jiang, Cheng-jun Li, Zheng-sheng Wu

**Affiliations:** ^1^ Department of Pathology, Anhui Medical University, Hefei, China; ^2^ Department of Pathology, The First Affiliated Hospital of Anhui Medical University, Hefei, China; ^3^ Zhejiang Provincial Key Laboratory of Pancreatic Disease, The First Affiliated Hospital, School of Medicine, Zhejiang University, Hangzhou, China; ^4^ The Key Laboratory of Developmental Genes and Human Disease, Institute of Life Sciences, Southeast University, Nanjing, China

**Keywords:** estrogen receptor, progesterone receptor, expression profile, pathological correlation, genetic alteration, clinical relevance, immunological correlation, survival contribution

## Abstract

**Introduction:**

Estrogen receptors (ESRs) and progesterone receptors (PGRs) are associated with the development and progression of various tumors. The feasibility of ESRs and PGRs as prognostic markers and therapeutic targets for multiple cancers was evaluated *via* pan-cancer analysis.

**Methods:**

The pan-cancer mRNA expression levels, genetic variations, and prognostic values of *ESR1*, *ESR2*, and *PGR* were analyzed using the Gene Expression Profiling Interactive Analysis 2 (GEPIA2) and cBioPortal. The expression levels of ERa, ERb, and PGR proteins were detected by immunohistochemical staining using paraffin-embedded tissue specimens of ovarian serous cystadenocarcinoma (OV) and uterine endometrioid adenocarcinoma (UTEA). Correlation between immunomodulators and immune cells was determined based on the Tumor and Immune System Interaction Database (TISIDB).

**Results:**

*ESR1*, *ESR2*, and *PGR* mRNAs were found to be differentially expressed in different cancer types, and were associated with tumor progression and clinical prognosis. ERa, ERb, and PGR proteins were further determined to be significantly differentially expressed in OV and UTEA *via* immunohistochemical staining. The expression of ERa protein was positively correlated with a high tumor stage, whereas the expression of PGR protein was conversely associated with a high tumor stage in patients with OV. In patients with UTEA, the expression levels of both ERa and PGR proteins were conversely associated with tumor grade and stage. In addition, the expression levels of *ESR1*, *ESR2*, and *PGR* mRNAs were significantly associated with the expression of immunomodulators and immune cells.

**Conclusion:**

*ESR1*, *ESR2*, and *PGR* are potential prognostic markers and therapeutic targets, as well as important factors for the prediction, evaluation, and individualized treatment in several cancer types.

## Introduction

Nowadays, cancer has become a leading cause of death worldwide, with continuously increasing rates of morbidity and mortality (1). In 2017, approximately 2.6 million Chinese individuals died of various types of cancer, accounting for 26.07% of the total deaths (2, 3). Multiple therapeutic strategies, including but not limited to surgery, radiotherapy, chemotherapy, and immunotherapy, have been developed for the comprehensive and individualized treatment of malignant tumors. However, overall clinical outcomes in patients with advanced cancers are still dissatisfactory, especially given the concomitant adverse effects. Therefore, there is an urgent need to identify potentially valuable molecular targets for the improvement of therapeutic efficacy and specificity.

Estrogen receptors (ESRs) belong to nuclear receptor superfamily of hormone-inducible transcription factors, which comprise ERa and ERb, encoded by *ESR1* and *ESR2*, respectively (4, 5). *PGR* encodes a member of the steroid receptor superfamily, named progesterone receptors (PGRs) (6). In physiological state, the activation of ESRs and PGRs by the binding of their ligands are associated with a series of normal physical activities. However, under pathological conditions, *ESR1*, *ESR2*, and *PGR* have been demonstrated to be associated with tumorigenesis and tumor progression (7, 8). For instance, *ESR1* is well characterized as a factor that promotes cell proliferation in breast cancer (9). In contrast, *ESR2* seems to be a tumor suppressor gene (10), which is not expressed in early stages of breast cancer (11). Further, *PGR* is associated with the development of breast cancer (12). In addition to breast cancer, *ESR1*, *ESR2*, and *PGR* also mediate the progression of prostate cancer (13–15), colon cancer (16–18), ovarian cancer (19–21), and lung cancer (22–24). Accordingly, *ESR1*, *ESR2*, and *PGR* may be prognostic biomarkers as well as potential therapeutic targets for a variety of cancer types, necessitating further evaluation.

In this study, we conducted a comprehensive pan-cancer analysis of *ESR1*, *ESR2*, and *PGR* on the basis of online databases. The expression levels of *ESR1*, *ESR2*, and *PGR*, and the correlation of *ESR1*, *ESR2*, and *PGR* with overall survival (OS) and disease-free survival (RFS) in patients were assessed using Gene Expression Profiling Interactive Analysis 2 (GEPIA2). The expression levels of ERa, ERb, and PGR proteins in ovarian serous cystadenocarcinoma (OV) and uterine endometrioid adenocarcinoma (UTEA) were validated using *in-house* tissue specimens, and the relationship between protein levels of ERa, ERb, and PGR and clinicopathological characteristics of OV or UTEA patients was explored. Genetic alterations and immunological effects of *ESR1*, *ESR2*, and *PGR* were analyzed using the cBioPortal and Tumor and Immune System Interaction Database (TISIDB), respectively.

## Materials and Methods

### Patient Tissue Sample Collection

Forty-two paraffin-embedded OV and 51 UTEA tissue specimens were collected from patients who underwent surgery at the High-tech district of the First Affiliated Hospital of Anhui Medical University (Hefei, Anhui, China) between 2017 and 2019. We also collected 11 specimens of normal ovarian tissue from 42 patients with OV (31 specimens of tumors involving bilateral ovarian tissue were excluded) and 34 specimens of normal endometrial tissue adjacent to the cancer in 51 patients with UTEA (17 specimens of tumors involving the entire endometrial tissue were excluded). No patient had a history of other malignant tumors and no patient had undergone preoperative interventions such as radiotherapy or chemotherapy. Each patient provided written informed consent, and the study was approved by the institutional review board.

### GEPIA2 Dataset Analysis

The expression levels of *ESR1*, *ESR2*, and *PGR* mRNAs in tumor and matched normal samples were compared using the GEPIA2 database, which is a webserver that provides cancer genomics data based on TCGA, and the GTEx database (25). In this study, differentially expressed gene analysis of tumor and matched normal samples, isoform profiling, and clinicopathological stage analysis were performed using the GEPIA2 dataset. Differentially expressed gene analysis and clinicopathological stage analysis were conducted by one-way ANOVA. Genes with |log2FC| > 1 and Q-value < 0.01 were considered to be differentially expressed. We used log_2_(TPM+1) for log-scaling differential expression in different clinicopathological stages, and regarded Pr(>F) < 0.05 to be statistically significant. In addition, correlative prognostic analysis of *ESR1*, *ESR2*, and *PGR*, including OS and RFS, was conducted to evaluate the prognostic significance using log-rank test for hypothesis evaluation at the median cutoff with 50% for either low- or high-expression cohorts.

### cBioPortal Analysis

The cBioPortal for Cancer Genomics is a widely used open-access website, providing a visualization and analysis tool for multidimensional cancer genomics data (26, 27). The cBioPortal was employed to analyze the OncoPrint, mutual exclusivity, alteration frequency in multiple cancer types, and amino acid changes in proteins and for the Clinical Attribute Test. Mutual exclusivity analysis among *ESR1*, *ESR2*, and *PGR* was conducted using Log_2_ odds ratio, P-value, and Q-value, and P-value < 0.001 and Q-value < 0.001 were regarded as statistically significant.

### TISIDB Analysis

The TISIDB is a user-friendly web portal containing 988 immune-related anti-tumor genes derived from 4,176 records in 2,530 publications. This database enables users to analyze the function of selected genes in the tumor–immune interplay through high-throughput data analysis or literature mining (28). In this study, we used TISIDB to construct heat maps for analyzing the spearman correlations between the expression levels of *ESR1*, *ESR2*, and *PGR* and immunomodulators and immune cells in multiple cancer types. A *p* value < 0.05 was regarded as statistically significant.

### Immunohistochemical Analysis

The *in situ* protein expression levels of ERa, ERb, and PGR in paraffin-embedded OV and UTEA tissue sections were detected by immunohistochemistry using rabbit polyclonal antibodies against ESR1 (1:200, 21244-1-AP, Proteintech), ESR2 (1:50, 14007-1-AP, Proteintech), and PGR (1:50, 25871-1-AP, Proteintech). Five fields were randomly observed at high power under the microscope. ERa, ERb, and PGR staining intensity of the tumor cells (0, no tumor cells stained yellow; 1, light yellow stain; 2, medium depth yellow stain; and 3, dark yellow stain) and the percentage of stained cells (0, no positive tumor cells; 1, <25% positive cells, 2, 25%–50% positive cells, and 3, > 50% positive cells) were recorded, and the sum of the two group scores ranged from 0 to 6 (17). Samples with staining scores of 0–3 were designated as ERa/ERb/PGR low expression, whereas those with staining scores >3 were designated as ERa/ERb/PGR high expression.

### Statistical Analysis

SPSS22.0 was used for data analysis. Chi-square test was used for variable comparison, with *p* < 0.05 regarded as statistically significant. Spearman’s method was used to assess the correlation between factors. *p* < 0.05 was regarded as statistically significant.

## Results

### *ESR1*, *ESR2*, and *PGR* mRNAs Are Differentially Expressed in Various Cancers

**Figure 1:**
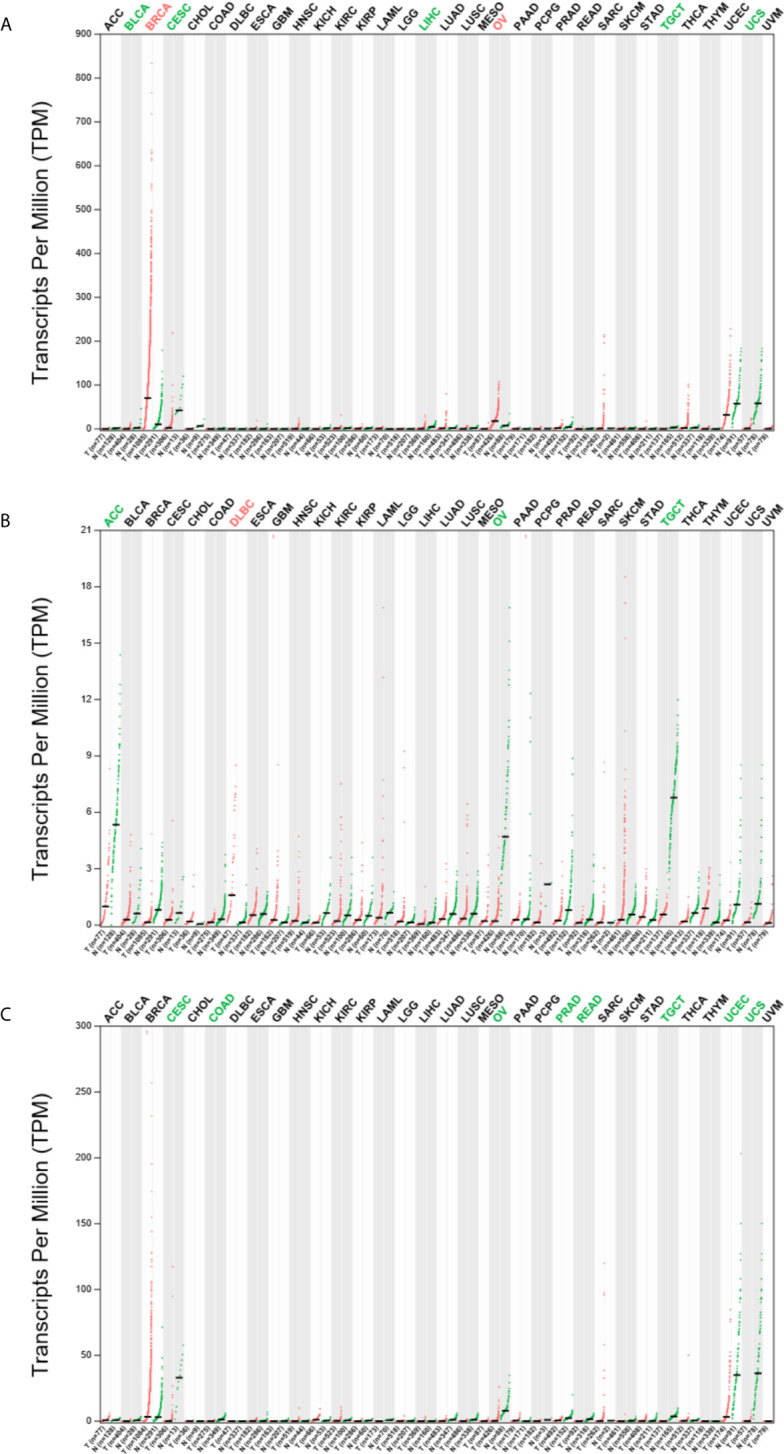
Expression profile of *ESR1*, *ESR2*, and *PGR* mRNAs across multiple cancer and matched normal samples. **(A)** Expression profile of *ESR1* mRNA across multiple cancer and matched normal samples. **(B)** Expression profile of *ESR2* mRNA across multiple cancer and matched normal samples. **(C)** Expression profile of *PGR* mRNA across multiple cancer and matched normal samples.

To explore the expression of ESR1, ESR2 and PGR in pan-cancer, we analyzed their mRNA levels *via* GEPIA2. We found that the *ESR1* mRNA was highly expressed in breast invasive carcinoma (BRCA) and OV samples compared with their matched normal samples (**Figure 1A**). In contrast, low expression levels of *ESR1* mRNA were found in bladder urothelial carcinoma (BLCA), cervical squamous cell carcinoma and endocervical adenocarcinoma (CESC), liver hepatocellular carcinoma (LIHC), testicular germ cell tumors (TGCT), and uterine carcinosarcoma (UCS) samples. *ESR2* mRNA was observed to be highly expressed only in the lymphoid neoplasm diffuse large B-cell lymphoma (DLBCL) samples, whereas a low expression of *ESR2* mRNA was found in adrenocortical carcinoma (ACC), OV, and TGCT samples (**Figure 1B**). In addition, a low expression of *PGR* mRNA was found in CESC, colon adenocarcinoma (COAD), OV, prostate adenocarcinoma (PRAD), rectal adenocarcinoma (READ), TGCT, UCEC, and UCS samples (**Figure 1C**). Together, *ESR1*, *ESR2*, and *PGR* are differentially expressed in multiple cancer types.

### *ESR1*, *ESR2*, and *PGR* Isoforms Are Differentially Expressed in Different Cancer Types

**Figure 2:**
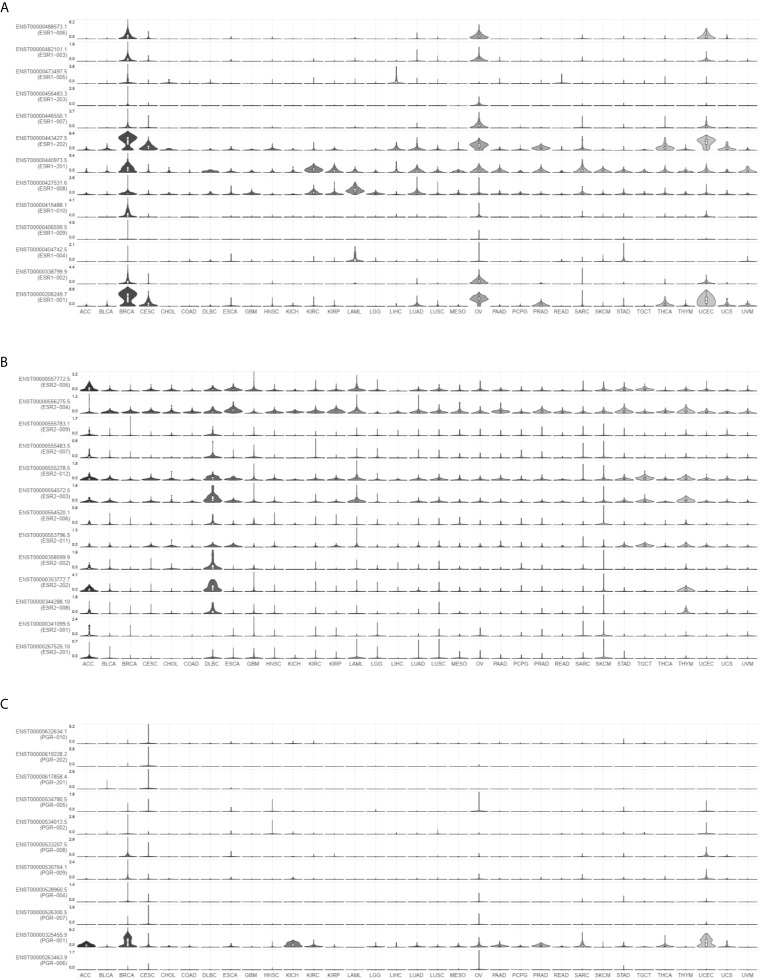
Expression distribution of *ESR1*, *ESR2*, and *PGR* mRNAs across multiple cancer types. **(A)** Expression distribution of *ESR1* mRNA across multiple cancer types. **(B)** Expression distribution of *ESR2* mRNA across multiple cancer types. **(C)** Expression distribution of *PGR* mRNA across multiple cancer types.

**Figure 3:**
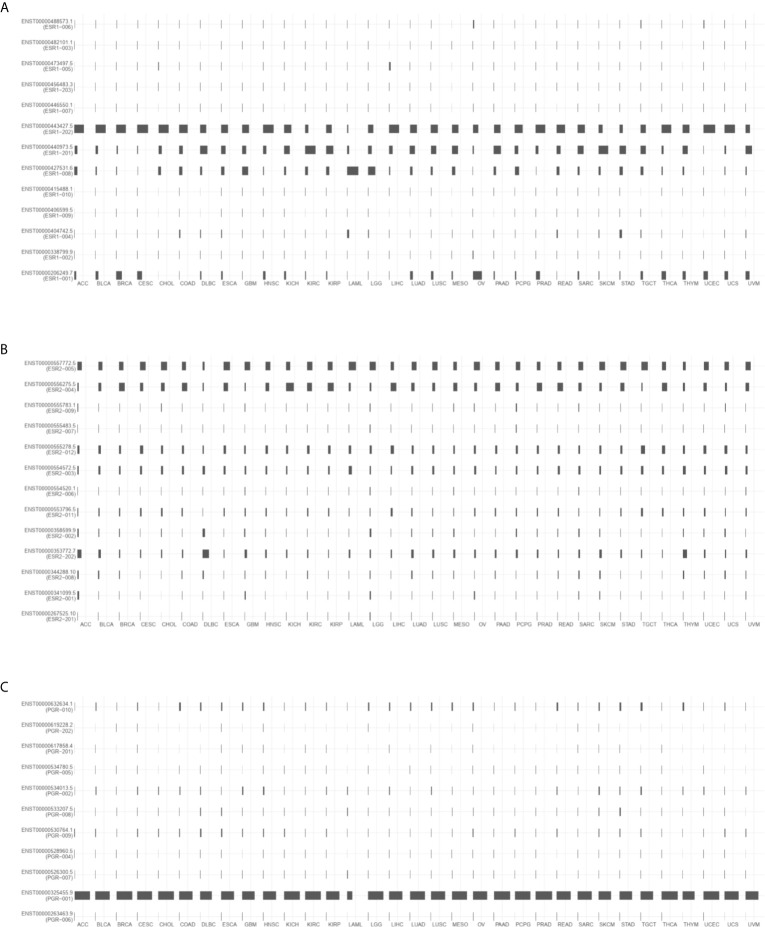
Isoform expression of *ESR1*, *ESR2*, and *PGR* genes across multiple cancer types. **(A)** Isoform expression of *ESR1* gene across multiple cancer types. **(B)** Isoform expression of *ESR2* gene across multiple cancer types. **(C)** Isoform expression of *PGR* gene across multiple cancer types.

To investigate the distribution of ESR1, ESR2, and PGR isoforms in pan-cancer, we compared their expression levels *via* GEPIA2. As shown in **Figures 2A–C**, the most prevalent transcripts are differentially expressed across multiple cancer types. For example, ESR-202 was the most prevalent *ESR1* transcript in BRCA samples, followed by ESR-001 and ESR-201, whereas ESR-004 was the most prevalent *ESR2* transcript in the same samples. In DLBCL samples, ESR-201 was the common *ESR1* transcript and ESR-202 was the most prevalent *ESR2* transcript. We also profiled isoform usage of these genes (**Figures 3A–C**). ESR1-201, ESR1-202, ESR2-004, ESR2-005 and PGR-001 were mostly commonly used transcribed isoforms in different cancer types. Thus, there exists isoform transformation during the transcription process of these genes as per the cancer type. Together, *ESR1*, *ESR2*, and *PGR* isoforms are differentially expressed in different cancer types.

### Correlation Between the Expression of *ESR1*, *ESR2*, and *PGR* mRNAs and Tumor Stage Across Multiple Cancers

**Figure 4:**
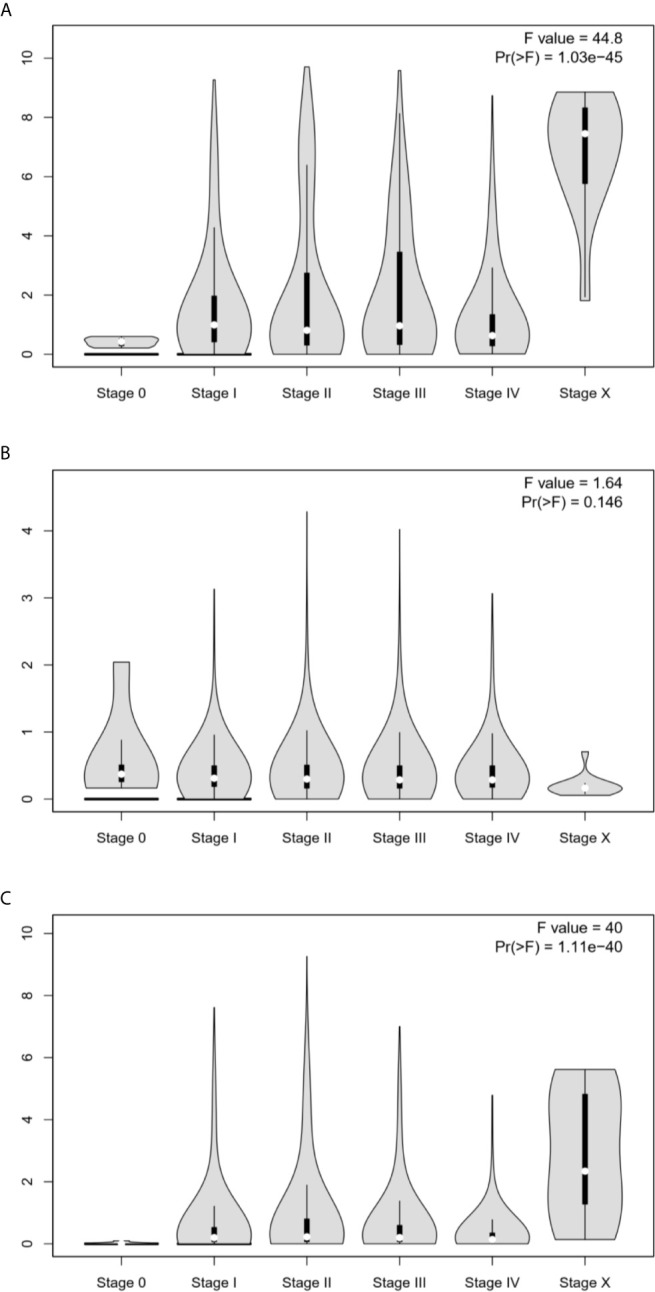
Correlation between *ESR1*, *ESR2*, and *PGR* mRNA expression levels and the tumor stage in multiple cancer samples. **(A)** Correlation between *ESR1* mRNA expression level and tumor stage in different cancer samples. **(B)** Correlation between *ESR2* mRNA expression level and tumor stage in different cancer samples. **(C)** Correlation between *PGR* mRNA expression level and tumor stage in different cancer samples.

To examine the clinical relevance of ESR1, ESR2, and PGR in pan-cancer, we analyzed the correlations of their expression with tumor stage. As shown in **Figures 4A–C**, *ESR1* and *PGR* transcription levels were correlated with the tumor stage (*p* < 0.05), and the higher expression of ESR1 and ESR2 are associated with higher tumor stage. In contrast, no significant association between *ESR2* and tumor stage was observed (*p* > 0.05). Taken together, the expression of ESR1 and PGR was significantly associated with pan-cancer tumor stage.

### Expression Levels of ERa, ERb, and PGR Proteins in OV and UTEA

**Figure 5:**
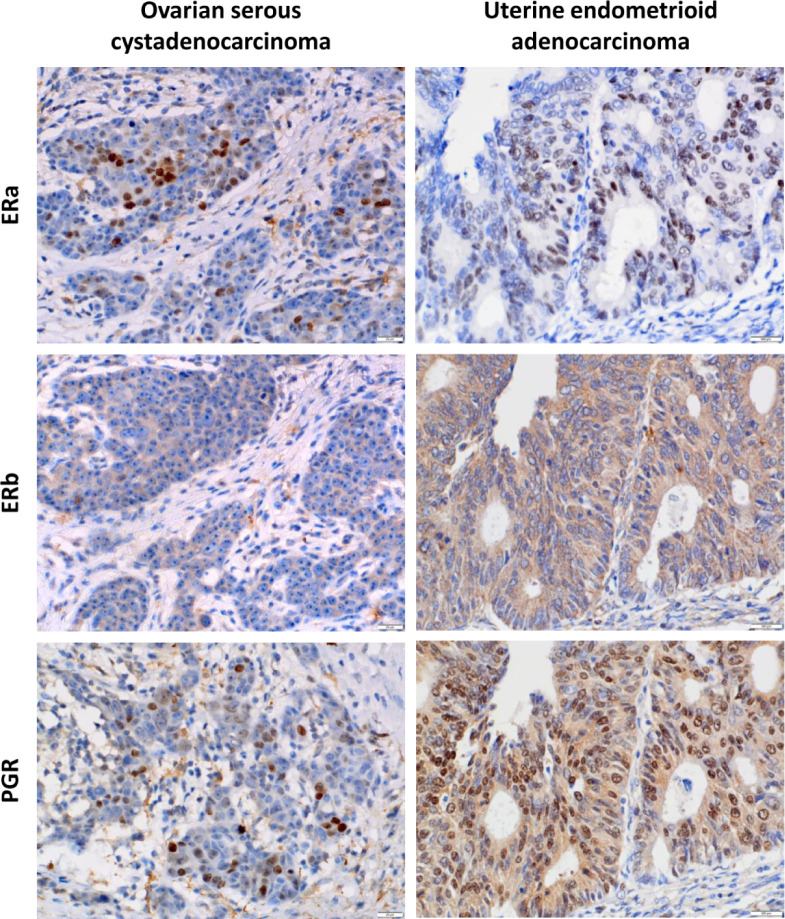
Immunohistochemical analysis of ERa, ERb, and PGR protein expression levels in ovarian serous cystadenocarcinoma and uterine endometrioid adenocarcinoma. Representative immunohistochemical images showed that ERa, ERb, and PGR were differentially expressed in ovarian serous carcinoma and endometrioid adenocarcinoma tissue. Left panels, low expression levels of ERa, ERb, and PGR proteins in serous ovarian carcinoma. Right panels, high expression levels of ERa, ERb, and PGR proteins in endometrioid adenocarcinoma. All micrographs were captured at ×400 magnification.

To detect the expression levels of ERa, ERb, and PGR proteins in OV and UTEA, which were not reported in any previous studies, we next performed immunohistochemical staining using paraffin-embedded tissue specimens. The results showed that the expression level of ERa was significantly higher, while ERb and PGR were significantly lower in OV compared to these in their matched normal samples (*p* < 0.05) (**Supplementary Material Figure S1A**). The expression levels of ERa, ERb, and PGR proteins were significantly lower in UTEA samples compared with these in their matched normal samples (*p* < 0.05) (**Supplementary Material Figure S1B**). Moreover, ERa, ERb, and PGR proteins were highly expressed in 42.9%, 50%, and 21.4% of OV tumor samples and in 64.7%, 35.3%, and 60.8% of UTEA tumor samples, respectively (**Table 1**). Together, the expression trends of ERa, ERb, and PGR proteins in OV and UTEA as well as the matched normal tissue are in consistent with the findings obtained from GEPIA2 (**Figure 5**).

**Table 1:** Association among ERa, ERb, and PGR protein expression levels in tumor tissues of patients with ovarian serous cystadenocarcinoma and uterine endometrioid adenocarcinoma.

Parameter	n	ERa		P Value	ERb		P Value	PGR		P Value
		Low expression	High expression		Low expression	High expression		Low expression	High expression	
**Ovarian serous cystadenocarcinoma**	42	24 (57.1%)	18 (42.9%)	**0.0351**	21 (50.0%)	21 (50.0%)	0.1526	33 (78.6%)	9 (21.4%)	**0.0001**
**Uterine endometrioid adenocarcinoma**	51	18 (35.3%)	33 (64.7%)		33 (64.7%)	18 (35.3%)	20 (39.2%)	31 (60.8%)

P value < 0.05 in the table was marked in bold, which was regarded as statistically significant.

### Association Between the Expressions of ERa, ERb, and PGR Proteins and the Clinicopathological Characteristics of Patients With OV or UTEA

To explore the clinical significance of ERa, ERb, and PGR expression in OV and UTEA, we further correlated their expression to clinicopathological characteristics of patients with OV or UTEA. Interestingly, the expression level of ERa protein was positively correlated to a high tumor stage, whereas the expression level of PGR protein was inversely correlated to a high tumor stage in patients with OV (Both *p* < 0.05) (**Table 2**). In UTEA, the expression levels of both ERa and PGR proteins were inversely correlated with high tumor grade and stage (all *p* < 0.05); this trend was not found in ERb (both *p* > 0.05) (**Table 3**). Further, the expression levels of ERb and PGR proteins were significantly correlated with patient age (both *p* < 0.05) (**Table 3**). Collectively, the expression of ERa, ERb, and PGR might be associated with the progression of OV and UTEA.

**Table 2:** Association of ERa, ERb, and PGR protein expression levels in tumors with the clinicopathological characteristics of patients with ovarian serous cystadenocarcinoma.

Parameter	n	ERa		P Value	ERb		P Value	PGR		P Value
		Low expression	High expression		Low expression	High expression		Low expression	High expression	
**Age (years)**				0.5329			0.7576			0.0601
** <60**	21	13 (61.9%)	8 (38.1%)		11 (52.4%)	10 (47.6%)		14 (66.7%)	7 (33.3%)	
**≥60**	21	11 (52.4%)	10 (47.6%)		10 (47.6%)	11 (52.4%)		19 (90.5%)	2 (9.5%)	
**Menopausal status**				0.1859			0.5126			0.1106
** Premenopausal**	14	10 (71.4%)	4 (28.6%)		8 (57.1%)	6 (42.9%)		9 (64.3%)	5 (35.7%)	
** Postmenopausal**	28	14 (50.0%)	14 (50.0%)		13 (46.4%)	15 (53.6%)		24 (85.7%)	4 (14.3%)	
**Lymph node metastasis**				0.7890			0.0637			0.5907
** +**	20	11 (55.0%)	9 (45.0%)		13 (65.0%)	7 (35.0%)		15 (75.0%)	5 (25.0%)	
**-**	22	13 (59.1%)	9 (40.9%)		8 (36.4%)	14 (63.7%)		18 (81.8%)	4 (18.2%)	
**Peritoneal implantation metastasis**				0.9136			0.0601			0.3947
** +**	33	19 (57.6%)	14 (42.4%)		14 (42.4%)	19 (57.6%)		25 (75.8%)	8 (24.2%)	
**-**	9	5 (55.6%)	4 (44.4%)		7 (77.8%)	2 (22.2%)		8 (88.9%)	1 (11.1%)	
**Stage**				**0.0002**			0.3456			**0.0183**
** Ⅰ+II+III**	17	13 (76.5%)	4 (23.5%)		10 (58.8%)	7 (41.2%)		6 (35.3%)	11 (64.7%)	
** Ⅳ**	25	5 (20.0%)	20 (80.0%)		11 (44.0%)	14 (56.0%)		18 (72.0%)	7 (28.0%)	

P value < 0.05 in the table was marked in bold, which was regarded as statistically significant.

**Table 3:** Association of ERa, ERb, and PGR protein expression levels in tumors with the clinicopathological characteristics of patients with uterine endometrioid adenocarcinoma.

Parameter	n	ERa		P Value	ERb		P Value	PGR		P Value
		Low expression	High expression		Low expression	High expression		Low expression	High expression	
**Age (yr)**				0.8500			**0.0341**			**0.0497**
** <60**	36	13 (36.1%)	23 (63.9%)		20 (55.6%)	16 (44.4%)		11 (30.6%)	25 (69.4%)	
** ≥60**	15	5 (33.3%)	10 (66.7%)		13 (86.7%)	2 (13.3%)		9 (60.0%)	6 (40.0%)	
**Menopausal status**				0.4649			0.8893			0.7163
** Premenopausal**	22	9 (40.9%)	13 (59.1%)		14 (63.6%)	8 (36.4%)		8 (36.4%)	14 (63.6%)	
** Postmenopausal**	29	9 (31.0%)	20 (69.0%)		19 (65.5%)	10 (34.5%)		12 (41.4%)	17 (58.6%)	
**Lymph node metastasis**				0.4259			0.4259			0.3596
** +**	11	5 (45.5%)	6 (54.5%)		6 (54.5%)	5 (45.5%)		3 (27.3%)	8 (72.7%)	
**-**	40	13 (32.5%)	27 (67.5%)		27 (67.5%)	13 (32.5%)		17 (42.5%)	23 (57.5%)	
**Cervical involvement**				0.0794			0.8869			0.4963
** Positive**	43	13 (30.2%)	30 (69.8%)		28 (65.1%)	15 (34.9%)		16 (37.2%)	27 (62.8%)	
** Negative**	8	5 (62.5%)	3 (37.5%)		5 (62.5%)	3 (37.5%)		4 (50.0%)	4 (50.0%)	
**Myometrial invasion**				0.5296			0.2287			0.8268
** <1/2**	40	15 (37.5%)	25 (62.5%)		25 (62.5%)	15 (37.5%)		16 (40.0%)	24 (60.0%)	
** ≥1/2**	11	3 (27.3%)	8 (72.7%)		9 (81.8%)	2 (18.2%)		4 (36.4%)	7 (63.6%)	
**Stage**				**0.0446**			0.0535			**0.0241**
** Ⅰ**	37	10 (27.0%)	27 (73.0%)		21 (56.8%)	16 (43.2%)		11 (29.7%)	26 (70.3%)	
** Ⅱ+Ⅲ**	14	8 (57.1%)	6 (42.9%)		12 (85.7%)	2 (14.3%)		9 (64.3%)	5 (35.7%)	
**Grade**				**0.0026**			0.0896			**0.0205**
** 1**	23	3 (13.0%)	20 (87.0%)		12 (52.2%)	11 (47.8%)		5 (21.7%)	18 (78.3%)	
** 2+3**	28	15 (53.6%)	13 (46.4%)		21 (75.0%)	7 (25.0%)		15 (53.6%)	13 (46.4%)	

P value < 0.05 in the table was marked in bold, which was regarded as statistically significant.

### Genetic Alterations and Clinical Relevance of *ESR1*, *ESR2*, and *PGR* in Different Cancers

**Figure 6:**
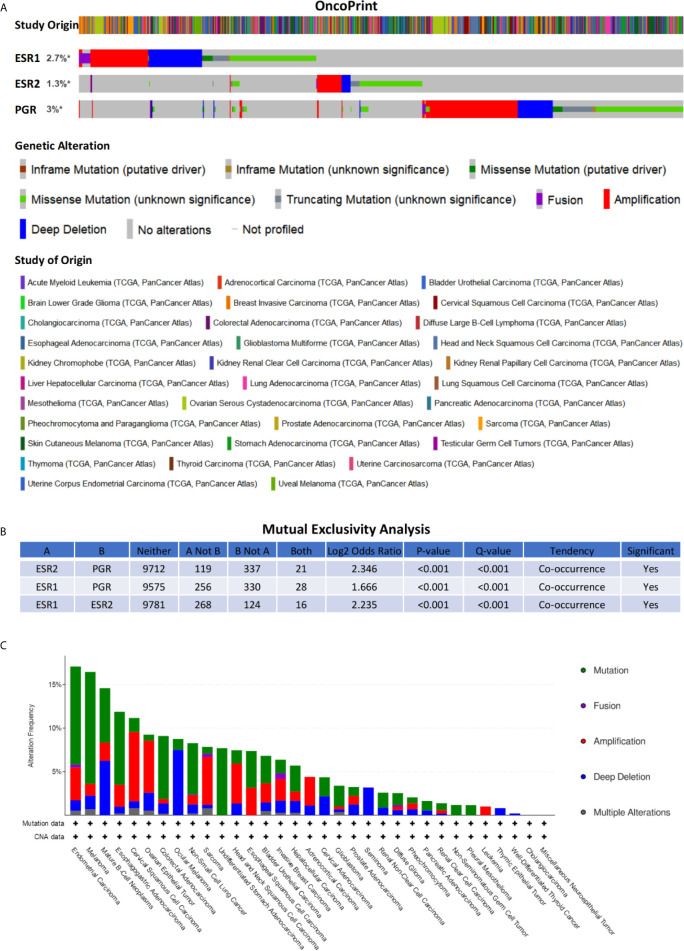
Genetic alterations of *ESR1*, *ESR2*, and *PGR* across multiple cancer types. **(A)** Alteration landscape for *ESR1*, *ESR2*, and *PGR* across multiple cancer types. **(B)** Mutual exclusivity analysis between alterations of *ESR1*, *ESR2*, and *PGR* across multiple cancer types. **(C)** Cancer type summary of *ESR1*, *ESR2*, and *PGR* alterations across multiple cancer types. * indicates not-profiled samples existing in the enquired gene.

**Figure 7:**
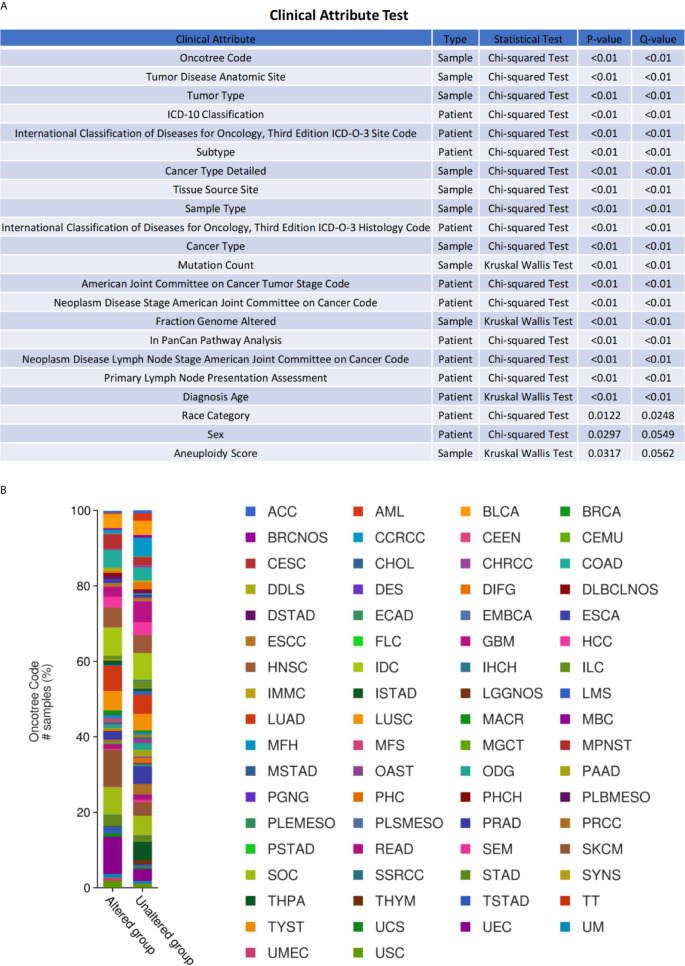
Clinical relevance of *ESR1*, *ESR2*, and *PGR* alterations across multiple cancer types. **(A)** OncoTree code of *ESR1*, *ESR2*, and *PGR* in different cancer types. **(B)** Clinical attribute test of *ESR1*, *ESR2*, and *PGR* in different cancer types.

To inquiry genetic alterations of *ESR1*, *ESR2*, and *PGR* that may be associated with tumorigenesis, we analyzed these in pan-cancer involving a total of 10,189 patients. Genetic alterations (including amplification, fusion, deep deletion, missense mutation, and truncating mutation) were detected in 2.7%, 1.3%, and 3% of *ESR1*, *ESR2*, and *PGR* genes, respectively (**Figure 6A**). Moreover, a mutual exclusivity analysis showed the selected genes tended toward co-occurrence rather than mutual exclusivity (*p* < 0.05) (**Figure 6B**). Mutations in *ESR1*, *ESR2*, and *PGR* genes were the most frequent alterations in multiple cancer types, followed by amplifications and deep deletions (**Figure 6C**). The patients were further divided into *ESR1*, *ESR2* and *PGR* altered and unaltered groups to conduct the clinical attribute test (**Figure 7A**). OncoTree Code was selected to indicate the ratio of cancer patients with/without genetic alterations in *ESR1*, *ESR2*, and *PGR* (**Figure 7B**), and results suggested the potentially critical roles of *ESR1*, *ESR2* and *PGR* in onset of multiple cancers. Together, *ESR1*, *ESR2*, and *PGR* are likely closely correlated and have a role in multiple tumor genesis.

### Mutation Site Analysis of *ESR1*, *ESR2*, and *PGR* in Multiple Cancer Types

**Figure 8:**
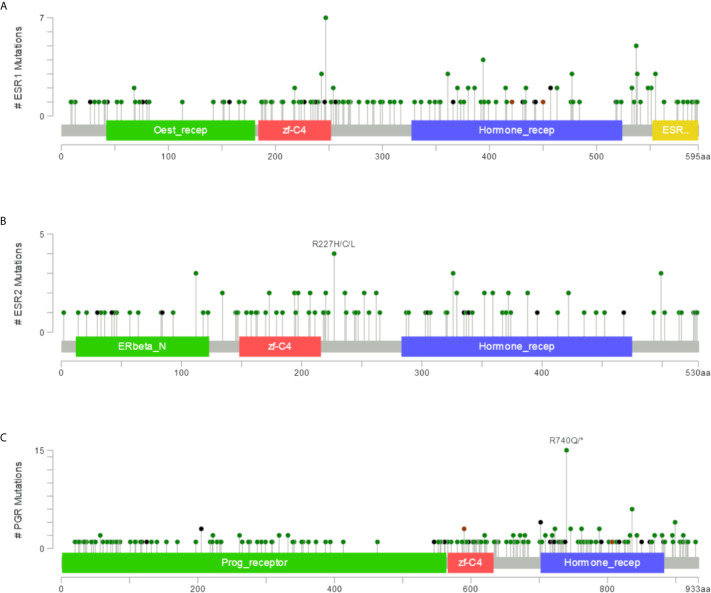
Mutations in *ESR1*, *ESR2*, and *PGR* across multiple cancer types. **(A)** Mutation frequency in *ESR1* across multiple cancer types. **(B)** Mutation frequency in *ESR2* across multiple cancer types. **(C)** Mutation frequency in *PGR* across multiple cancer types.

To identify mutation sites in the *ESR1*, *ESR2*, and *PGR* genes, we assessed 10,189 samples from multiple cancer types. The mutation sites were most commonly located within the Oest_recep, zf-C4, Hormone_recep, and ESR1_C domains (**Figure 8A**). Specifically, 169 mutations of *ESR1* were detected, consisting of 131 missense mutations, 19 truncating mutations, 3 inframe mutations, and 16 other types of mutations. Seven of these mutations were E247K/D, a hotspot for protein activation. In addition, 112 *ESR2* and 214 *PGR* nonsynonymous mutation sites were detected in different cancers, with the highest frequency mutations in R227H/C/L and R740Q/* (**Figures 8B, C**). Together, there are an abundance of mutation sites of *ESR1*, *ESR2* and *PGR*, suggesting the complexity of their mutations.

### Immunological Correlation Between *ESR1*, *ESR2*, and *PGR* and Immune Modulatory Factors Across Multiple Cancer Types

**Figure 9:**
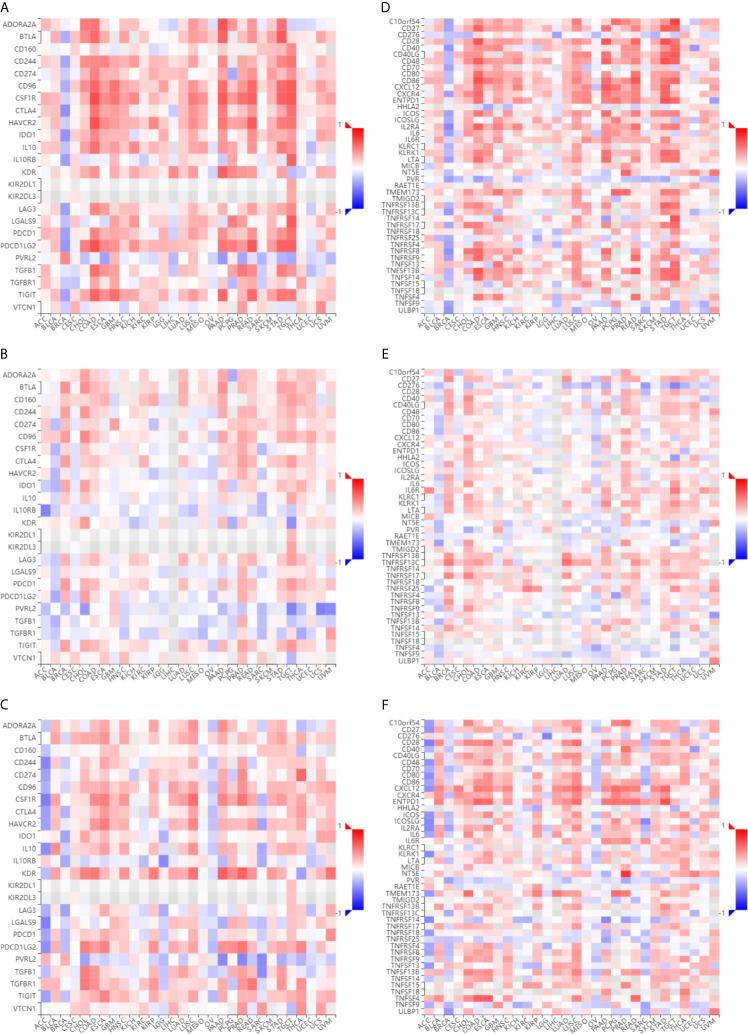
Immunological correlation of *ESR1*, *ESR2*, and *PGR* with various cancer immunomodulatory factors. **(A)** Correlations between *ESR1* and immunoinhibitors across multiple cancer types. **(B)** Correlations between *ESR2* and immunoinhibitors across multiple cancer types. **(C)** Correlations between *PGR* and immunoinhibitors across multiple cancer types. **(D)** Correlations between *ESR1* and immunostimulators across multiple cancer types. **(E)** Correlations between *ESR2* and immunostimulators across multiple cancer types. **(F)** Correlations between *PGR* and immunostimulators across multiple cancer types.

To assess the relevance of *ESR1*, *ESR2*, and *PGR* with immune system that plays critical roles in cancer progression (28), we first compared their co-expression with the abundance of immunomodulators. As shown in **Figures 9A–C**, there is a positive correlation of the expression levels of *ESR1*, *ESR2*, and *PGR* genes with multiple immune-inhibitors (such as CD274, TIGIT, and CTLA4). Moreover, the expression levels of *ESR1*, *ESR2*, and *PGR* were observed to have a positive correlation with several immune-stimulators (such as CD27, CD28, and CXCL12) (**Figures 9D–F**). These findings suggest *ESR1*, *ESR2* and *PGR* might be associate with both immune stimulation and inhibition. Together, the potential roles of *ESR1*, *ESR2* and *PGR* in cancer are likely in an immune system-dependent manner.

### Blood Cell Type-Specific Expression Profiles of *ESR1*, *ESR2*, and *PGR* Across Multiple Cancer Types

**Figure 10:**
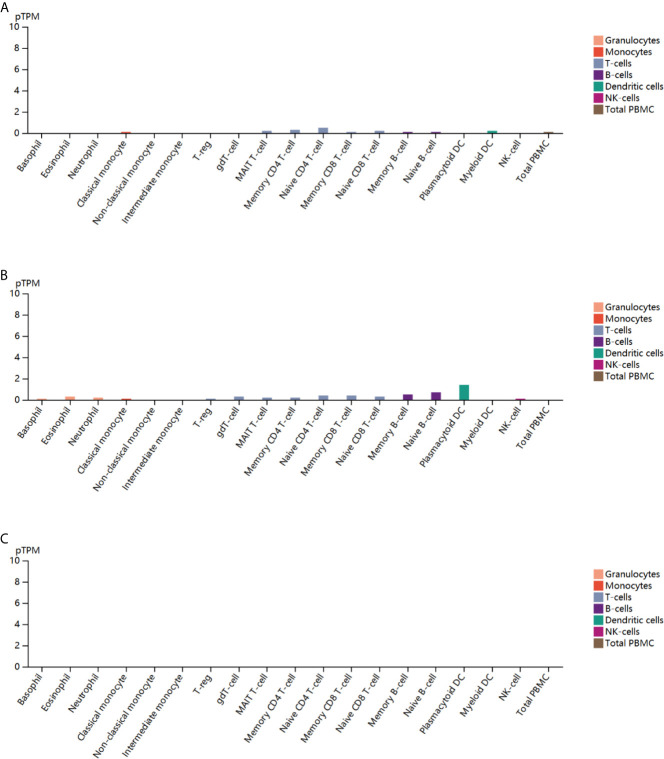
Blood cell type-specific expression profile of *ESR1*, *ESR2*, and *PGR* across multiple cancer types. **(A)** Blood cell type-specific distribution of *ESR1* across multiple cancer types. **(B)** Blood cell type-specific distribution of *ESR2* across multiple cancer types. **(C)** Blood cell type-specific distribution of *PGR* across multiple cancer types.

To further explore the correlation of *ESR1*, *ESR2* and *PGR* with immunity, we analyzed their expression in peripheral blood cell types. *ESR1* was found to be expressed in classical monocyte, MAIT T-cell, naive CD4 T cells, memory CD4 T cells, memory CD8 T-cell, naïve CD8 T cell, memory B cell and myeloid dendritic cells (DC) (**Figure 10A**). Similarly, the expression of *ESR2* was observed in multiple peripheral blood cells, with the highest expression level in plasma cell-like DC (**Figure 10B**). In contrast, *PGR* expression was not observed in peripheral blood cells (**Figure 10C**). Together, *ESR1* and *ESR2* might potentially influence multiple immune cells.

### Contribution of *ESR1*, *ESR2*, and *PGR* to Survival Across Multiple Cancer Types

**Figure 11:**
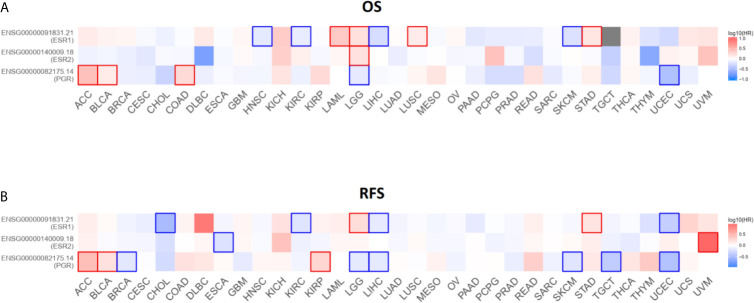
Survival contribution of *ESR1*, *ESR2*, and *PGR* across multiple cancer types. **(A)** Contribution analysis of *ESR1*, *ESR2*, and *PGR* to OS in multiple cancer types. **(B)** Contribution analysis of *ESR1*, *ESR2*, and *PGR* to RFS in multiple cancer types.

To investigate the potential roles of *ESR1*, *ESR2*, and *PGR* in prognosis, we analyzed the correlation of their expression with pan-cancer survival. As excepted, the expression of these genes was significantly associated with the OS and RFS in several cancers (**Figures 11A, B**). For instance, the higher expression of ESR1 is significantly related with superior OS in head and neck squamous cell carcinoma (HNSC), kidney renal clear cell carcinoma (KIRC), liver hepatocellular carcinoma (LIHC) and skin cutaneous melanoma (SKCM). In contrast, the down-regulation of ESR1 is suggestive of better OS in acute myeloid leukemia (LAML), brain lower grade glioma (LGG), lung squamous cell carcinoma (LUSC) and stomach adenocarcinoma (STAD). Together, these findings suggest that ESR1, ESR2 and PGR are potential prognostic factor in multiple cancers.

## Discussion

*ESRs* and *PGR* promote cell proliferation in breast cancer (29). Further, *ESRs* and *PGR*, which are associated with tumorigenesis and progression under pathological conditions, have become ideal molecular treatment targets (30–34). Accordingly, previous studies have demonstrated that drugs targeting *ESR1*, *ESR2*, and *PGR* are effective in the treatment of breast cancer and improve promote clinical outcomes (**Supplementary Material Tables S1–S3**). In addition, it has already been described the role of *ESR1*, *ESR2*, and *PGR* in promoting ovarian, lung, and prostate tumorigenesis (35–37). However, the roles of *ESR1*, *ESR2*, and *PGR* in other cancer types have rarely been studied and further investigations are needed to reach a consensus. In our study, we selected *ESR1*, *ESR2*, and *PGR* for an in-depth analysis of mRNA expression, genetic alternations, and clinical outcomes as well as the co-expression of these genes with immunomodulatory factors in a variety of cancer types. We also validated the expression levels of ERa, ERb, and PGR proteins in OV and UTEA using paraffin-embedded tissue specimens, and explored the relationship between ERa, ERb, and PGR proteins and clinicopathological characteristics of patients. To the best of our knowledge, this is the first such study based on integrated bioinformatics analysis. Through this comprehensive pan-cancer analysis, the feasibility of using *ESR1*, *ESR2*, and *PGR* as prognostic markers and therapeutic targets for multiple cancers was evaluated.

The results based on GEPIA2 dataset analysis were partly consistent with those reported previously (38–40). Hishida et al. showed that *ESR1* gene transcripts were absent or decreased in more than 90% of liver cancer (n = 24) samples compared with their matched normal liver tissue counterparts. These results highlighted *ESR1* as a tumor suppressor gene in liver cancer, and indicated that lower cellular estrogen levels stimulated liver cancer cell growth (41). The results of the present study revealed that the expression of *ESR1* and *PGR* correlated with the tumor stage, whereas the expression of *ESR2* did not. Additionally, prognostic analysis suggested that *ESR1*, *ESR2*, and *PGR* were significantly correlated with OS and RFS in patients with specific cancer types. However, due to the heterogeneity, subtypes, and sample size of cancers, or limited length of follow-up in TCGA datasets, there were discrepancies between the results of this study and those of previously published studies. For example, analysis using TCGA database showed that patients had shorter OS and RFS with higher ERb expression levels in renal cell carcinoma (42). This is in contrast to the results of the present study, which showed that *ESR2* expression was not a risk factor for kidney cancer. Generally, the results of this study indicated that *ESR1*, *ESR2*, and *PGR* can be regarded as predictive and prognostic biomarkers across different cancer types.

The progression of cancer is influenced by multiple factors including, but not limited to, somatically acquired genetic, epigenetic, transcriptomic and proteomic alterations (43). Some alterations in particular genomic regions exhibit potential pro- and anti-tumor effects (44). Therefore, we used the cBioPortal web tool for further analysis of the genetic mutations in *ESR1*, *ESR2*, and *PGR* in multiple cancer types. Our results revealed genetic alterations in *ESR1*, *ESR2*, and *PGR* in multiple cancer types, including amplification, fusion, deep deletion, missense mutation, and truncating mutation. In addition, we identified a trend for co-occurrence of genetic alterations in *ESR1*, *ESR2*, and *PGR*. Based on these results, combining the expressions of *ESR1*, *ESR2*, and *PGR* may provide a better prognostic value in cancer patients. Yi et al. proposed that higher *ESR1* expression and a higher ESR ratio (*ESR1/ESR2*) were associated with worse overall survival in female papillary thyroid carcinoma patients (45). There were also differences in the types and frequencies of genetic alterations in *ESR1*, *ESR2*, and *PGR* in multiple cancer types. Furthermore, mutations in *ESR1*, *ESR2*, and *PGR* could result in the amino acid changes in several sites. Considering these results, we hypothesized that genetic alterations in *ESR1*, *ESR2*, and *PGR* play an essential role in cancer progression and combining the expression levels of *ESR1*, *ESR2*, and *PGR* provide prognostic value.

It has been reported that genomic diversity increases with the rate of genetic alterations result in cancers, resulting in an increased frequency of neoantigens and greater immune cell infiltration (46). Understanding the effects of immune cells on cancers will lead to a new era in oncotherapy. Therefore, the effectiveness and efficiency of immune checkpoint-target agents, which direct the host immune system to target cancer cells, has become a focus of research. However, results showed relatively low response rates of immune checkpoint-target agents in some tumors (47–49). To overcome this challenge, further understanding of immunotherapy is needed to select the patients who will benefit most from this type of therapy. Studies have found that immune checkpoint proteins (PD-L1, VISTA) are more frequently expressed in certain *ESR*-negative breast cancers (50, 51). Liu et al. demonstrated an inverse correlation between *ESRs* and *PD-L1* in breast cancer cells, indicating that *PD-L1* gene transcription is negatively regulated by *ESRs* (52), which is consistent with the results of the current study. Hence, in the present study, we investigated the potential of *ESR1*, *ESR2*, and *PGR* as predictive and prognostic biomarkers in multiple cancer types from an immuno-oncological perspective based on bioinformatics analysis to provide a reference for future studies and the application of immunotherapies. In this study, we explored the relationship between *ESR1*, *ESR2*, and *PGR* and immunomodulators or immune cells using the TISIDB database. The results demonstrated that *ESR1* had the greatest correlation with immunoinhibitors (such as CD274, CD96, CFS1R, and CTLA-4) and immunostimulators (such as CD27, CD28, and CXCL12). It is noteworthy that the role of *ESR1* in the function of immunomodulators is context-dependent. *ESR2* and *PGR* showed similar results. In addition, we found that there was a certain relationship between the expression levels of *ESR1*, *ESR2*, and *PGR* and peripheral blood cells. Therefore, this preliminary analysis of the association between *ESR1*, *ESR2*, and *PGR* and immune function highlights the importance of future research to elucidate the potential roles of *ESR1*, *ESR2*, and *PGR* as predictive and prognostic biomarkers as well as therapeutic targets for immunotherapy across multiple cancer types.

Our study also has some limitations. The results derived from different online databases are inevitably accompanied by background heterogeneity. Moreover, our immunohistochemical verification experiment was conducted only in OV and UTEA, with inadequate prognostic studies of patient cohorts. More cancer types need to be included in subsequent verification experiments, which can be further verified by adding cytological function studies and patient cohort studies.

## Conclusions

In summary, we identified significant differences in the expression levels of *ESR1*, *ESR2*, and *PGR* mRNAs in different cancer types, which associated with tumor progression and clinical prognosis. Our study provides comprehensive evidence that *ESR1*, *ESR2*, and *PGR* are feasible prognostic markers and therapeutic targets for multiple cancers and that they could be a factor for disease prediction, disease evaluation, and individualized treatment in various types of cancer.

## Data Availability Statement

The datasets presented in this study can be found in online repositories. The names of the repository/repositories and accession number(s) can be found in the article/**Supplementary Material**.

## Ethics Statement

The studies involving human participants were reviewed and approved by Anhui Medical University, Hefei 230032, Anhui, China. The patients/participants provided their written informed consent to participate in this study.

## Author Contributions

XH and Z-sW conceived the study. Y-tS, XH, GZ, BJ, and C-jL collected the data. XH and Z-sW analyzed and interpreted the data. Y-tS, XH, and GZ wrote and revised the manuscript. All authors discussed and revised the manuscript. All authors contributed to the article and approved the submitted version. XH, Y-tS, and GZ contributed equally to the study. XH and Z-sW supervised the study and share the senior authorship.

## Funding

This study was funded by grants from the National Natural Science Foundation of China (31970696 and 81502975 to XH; 81972472 to Z-sW), China Postdoctoral Science Foundation (2016T90413 and 2015M581693 to XH), and Natural Science Foundation of Anhui (2008085MH276 to Z-sW).

## Conflict of Interest

The authors declare that the research was conducted in the absence of any commercial or financial relationships that could be construed as a potential conflict of interest.

## Abbreviations

ESR1, estrogen receptor 1; ESR2, estrogen receptor 2; PGR, progesterone receptor; GEPIA2, the Gene Expression Profiling Interactive Analysis2; TISIDB, the Tumor and Immune System Interaction Database; OS, overall survival; RFS, disease-free survival; BRCA, breast invasive carcinoma; OV, ovarian serous cystadenocarcinoma; UTEA, uterine endometrioid adenocarcinoma; BLCA, bladder urothelial carcinoma; CESC, cervical squamous cell carcinoma and endocervical adenocarcinoma; LIHC, liver hepatocellular carcinoma; TGCT, testicular germ cell tumors; UCS, uterine carcinosarcoma; DLBCL, lymphoid neoplasm diffuse large B-cell lymphoma; ACC, adrenocortical carcinoma; COAD, colon adenocarcinoma; PRAD, prostate adenocarcinoma; READ, rectum adenocarcinoma; UCEC, uterine corpus endometrial carcinoma; DC, dendritic cells; HNSC, neck squamous cell carcinoma; KIRC, kidney renal clear cell carcinoma; LIHC, liver hepatocellular carcinoma; SKCM, skin cutaneous melanoma; LAML, acute myeloid leukemia; LGG, brain lower grade glioma; LUSC, lung squamous cell carcinoma; STAD, stomach adenocarcinoma.

## References

[1] Feng RM Zong YN Cao SM Xu RH. Current Cancer Situation in China: Good or Bad News From the 2018 Global Cancer Statistics? Cancer Commun (Lond) (2019) 39:22. 10.1186/s40880-019-0368-6 31030667 PMC6487510

[2] Chen WQ Xia CF Zheng RS Zhou MG Lin CQ Zeng HM. Disparities by Province, Age, and Sex in Site-Specific Cancer Burden Attributable to 23 Potentially Modifiable Risk Factors in China: A Comparative Risk Assessment. Lancet Global Health (2019) 7:e257–69. 10.1016/s2214-109x(18)30488-1 30683243

[3] GBD 2017 Causes of Death Collaborators. Global, Regional, and National Age-Sex-Specific Mortality for 282 Causes of Death in 195 Countries and Territories, 1980–2017: A Systematic Analysis for the Global Burden of Disease Study 2017. Lancet (2018) 392:1736–88. 10.1016/s0140-6736(18)32203-7 PMC6227606 30496103

[4] Gibson DA Saunders PT. Estrogen Dependent Signaling in Reproductive Tissues - a Role for Estrogen Receptors and Estrogen Related Receptors. Mol Cell Endocrinol (2012) 348:361–72. 10.1016/j.mce.2011.09.026 21964318

[5] Hewitt SC Winuthayanon W Korach KS. What's New in Estrogen Receptor Action in the Female Reproductive Tract. J Mol Endocrinol (2016) 56:R55–71. 10.1530/JME-15-0254 PMC4733493 26826253

[6] Graham JD Clarke CL. Physiological Action of Progesterone in Target Tissues. Endocr Rev (1997) 18:502–19. 10.1210/edrv.18.4.0308 9267762

[7] Edwards DP. Regulation of Signal Transduction Pathways by Estrogen and Progesterone. Annu Rev Physiol (2005) 67:335–76. 10.1146/annurev.physiol.67.040403.120151 15709962

[8] Allison KH Hammond MEH Dowsett M McKernin SE Carey LA Fitzgibbons PL. Estrogen and Progesterone Receptor Testing in Breast Cancer: ASCO/CAP Guideline Update. J Clin Oncol (2020) 38:1346–66. 10.1200/JCO.19.02309 31928404

[9] Yager JD Davidson NE. Estrogen Carcinogenesis in Breast Cancer. N Engl J Med (2006) 354:270–82. 10.1056/NEJMra050776 16421368

[10] Rizza P Barone I Zito D Giordano F Lanzino M De Amicis F. Estrogen Receptor Beta as a Novel Target of Androgen Receptor Action in Breast Cancer Cell Lines. Breast Cancer Res (2014) 16:R21. 10.1186/bcr3619 24552459 PMC3978907

[11] Huang B Omoto Y Iwase H Yamashita H Toyama T Coombes RC. Differential Expression of Estrogen Receptor α, β1, and β2 in Lobular and Ductal Breast Cancer. Proc Natl Acad Sci (2014) 111:1933–8. 10.1073/pnas.1323719111 PMC3918808 24449868

[12] Cui X Schiff R Arpino G Osborne CK Lee AV. Biology of Progesterone Receptor Loss in Breast Cancer and its Implications for Endocrine Therapy. J Clin Oncol (2005) 23:7721–35. 10.1200/JCO.2005.09.004 16234531

[13] Cheng J Lee EJ Madison LD Lazennec G. Expression of Estrogen Receptor Beta in Prostate Carcinoma Cells Inhibits Invasion and Proliferation and Triggers Apoptosis. FEBS Lett (2004) 566:169–72. 10.1016/j.febslet.2004.04.025 15147889

[14] Prins GS Korach KS. The Role of Estrogens and Estrogen Receptors in Normal Prostate Growth and Disease. Steroids (2008) 73:233–44. 10.1016/j.steroids.2007.10.013 PMC2262439 18093629

[15] Grindstad T Richardsen E Andersen S Skjefstad K Rakaee Khanehkenari M Donnem T. Progesterone Receptors in Prostate Cancer: Progesterone Receptor B Is the Isoform Associated With Disease Progression. Sci Rep (2018) 8:11358. 10.1038/s41598-018-29520-5 30054508 PMC6063894

[16] Liu S Fan W Gao X Huang K Ding C Ma G. Estrogen Receptor Alpha Regulates the Wnt/Beta-Catenin Signaling Pathway in Colon Cancer by Targeting the NOD-Like Receptors. Cell Signal (2019) 61:86–92. 10.1016/j.cellsig.2019.05.009 31121307

[17] Peng J Ou Q Wu X Zhang R Zhao Q Jiang W. Expression of Voltage-Gated Sodium Channel Nav1.5 in Non-Metastatic Colon Cancer and Its Associations With Estrogen Receptor (ER)-Beta Expression and Clinical Outcomes. Chin J Cancer (2017) 36:89. 10.1186/s40880-017-0253-0 29122010 PMC5679318

[18] Slattery ML Samowitz WS Holden JA. Estrogen and Progesterone Receptors in Colon Tumors. Am J Clin Pathol (2000) 113:364–8. 10.1309/5MHB-K6XX-QV50-PCJQ 10705816

[19] Deroo BJ Korach KS. Estrogen Receptors and Human Disease. J Clin Invest (2006) 116:561–70. 10.1172/JCI27987 PMC2373424 16511588

[20] Treeck O Pfeiler G Mitter D Lattrich C Piendl G Ortmann O. Estrogen Receptor {Beta}1 Exerts Antitumoral Effects on SK-OV-3 Ovarian Cancer Cells. J Endocrinol (2007) 193:421–33. 10.1677/JOE-07-0087 17535880

[21] Pedernera E Gomora MJ Morales-Vasquez F Perez-Montiel D Mendez C. Progesterone Reduces Cell Survival in Primary Cultures of Endometrioid Ovarian Cancer. J Ovarian Res (2019) 12:15. 10.1186/s13048-019-0486-4 30736825 PMC6367846

[22] Hiramitsu S Ishikawa T Lee WR Khan T Crumbley C Khwaja N. Estrogen Receptor Beta-Mediated Modulation of Lung Cancer Cell Proliferation by 27-Hydroxycholesterol. Front Endocrinol (Lausanne) (2018) 9:470. 10.3389/fendo.2018.00470 30190703 PMC6116707

[23] Rades D Setter C Dahl O Schild SE Noack F. The Prognostic Impact of Tumor Cell Expression of Estrogen Receptor-Alpha, Progesterone Receptor, and Androgen Receptor in Patients Irradiated for Nonsmall Cell Lung Cancer. Cancer (2012) 118:157–63. 10.1002/cncr.26282 21713768

[24] Kawprasertsri S Pietras RJ Marquez-Garban DC Boonyaratanakornkit V. Progesterone Receptor (PR) Polyproline Domain (PPD) Mediates Inhibition of Epidermal Growth Factor Receptor (EGFR) Signaling in non-Small Cell Lung Cancer Cells. Cancer Lett (2016) 374:279–91. 10.1016/j.canlet.2016.02.014 PMC5708136 26892043

[25] Tang Z Kang B Li C Chen T Zhang Z. GEPIA2: An Enhanced Web Server for Large-Scale Expression Profiling and Interactive Analysis. Nucleic Acids Res (2019) 47:W556–60. 10.1093/nar/gkz430 PMC6602440 31114875

[26] Cerami E Gao J Dogrusoz U Gross BE Sumer SO Aksoy BA. The Cbio Cancer Genomics Portal: An Open Platform for Exploring Multidimensional Cancer Genomics Data. Cancer Discovery (2012) 2:401–4. 10.1158/2159-8290.CD-12-0095 PMC3956037 22588877

[27] Gao J Aksoy BA Dogrusoz U Dresdner G Gross B Sumer SO. Integrative Analysis of Complex Cancer Genomics and Clinical Profiles Using the Cbioportal. Sci Signaling (2013) 6:pl1. 10.1126/scisignal.2004088 PMC4160307 23550210

[28] Ru B Wong CN Tong Y Zhong JY Zhong SSW Wu WC. TISIDB: An Integrated Repository Portal for Tumor-Immune System Interactions. Bioinformatics (2019) 35:4200–2. 10.1093/bioinformatics/btz210 30903160

[29] White MM Zamudio S Stevens T Tyler R Lindenfeld J Leslie K. Estrogen, Progesterone, and Vascular Reactivity: Potential Cellular Mechanisms. Endocr Rev (1995) 16:739–51. 10.1210/edrv-16-6-739 8747833

[30] DeCensi A Pruneri G Guerrieri-Gonzaga A. Estrogen Receptor in Breast Ductal Carcinoma in Situ: Good Cop, Bad Cop? J Clin Oncol (2012) 30:1384–6. 10.1200/JCO.2011.40.7494 22393083

[31] Brisken C. Progesterone Signalling in Breast Cancer: A Neglected Hormone Coming Into the Limelight. Nat Rev Cancer (2013) 13:385–96. 10.1038/nrc3518 23702927

[32] Tanos T Sflomos G Echeverria PC Ayyanan A Gutierrez M Delaloye JF. Progesterone/RANKL Is a Major Regulatory Axis in the Human Breast. Sci Transl Med (2013) 5:182ra155. 10.1126/scitranslmed.3005654 23616122

[33] Jordan VC O'Malley BW. Selective Estrogen-Receptor Modulators and Antihormonal Resistance in Breast Cancer. J Clin Oncol (2007) 25:5815–24. 10.1200/JCO.2007.11.3886 17893378

[34] Fabian CJ Kimler BF. Selective Estrogen-Receptor Modulators for Primary Prevention of Breast Cancer. J Clin Oncol (2005) 23:1644–55. 10.1200/JCO.2005.11.005 15755972

[35] Smida T Bruno TC Stabile LP. Influence of Estrogen on the NSCLC Microenvironment: A Comprehensive Picture and Clinical Implications. Front Oncol (2020) 10:137. 10.3389/fonc.2020.00137 32133288 PMC7039860

[36] He M Yu W Chang C Miyamoto H Liu X Jiang K. Estrogen Receptor Alpha Increases Lung Cancer Cell Invasion via Increase of and Cross-Talk With Infiltrated Macrophages Through the CCL2/CCR2/MMP9 and CXCL12/CXCR4 Signaling Pathways. Mol Oncol (2020) 14(8):1779–99. 10.1002/1878-0261.12701 PMC7400793 32356397

[37] Warner M Huang B Gustafsson JA. Estrogen Receptor Beta as a Pharmaceutical Target. Trends Pharmacol Sci (2017) 38:92–9. 10.1016/j.tips.2016.10.006 27979317

[38] Salvati A Gigantino V Nassa G Giurato G Alexandrova E Rizzo F. The Histone Methyltransferase DOT1L Is a Functional Component of Estrogen Receptor Alpha Signaling in Ovarian Cancer Cells. Cancers (Basel) (2019) 11(11):1720. 10.3390/cancers11111720 PMC6895927 31689915

[39] Hirvonen-Santti SJ Rannikko A Santti H Savolainen S Nyberg M Jänne OA. Down-Regulation of Estrogen Receptor Beta and Transcriptional Coregulator SNURF/RNF4 in Testicular Germ Cell Cancer. Eur Urol (2003) 44:742–747; discussion 747. 10.1016/s0302-2838(03)00382-8 14644130

[40] Zhao Y Li Z. Interplay of Estrogen Receptors and FOXA Factors in the Liver Cancer. Mol Cell Endocrinol (2015) 418 Pt 3:334–9. 10.1016/j.mce.2015.01.043 PMC4524798 25661537

[41] Hishida M Nomoto S Inokawa Y Hayashi M Kanda M Okamura Y. Estrogen Receptor 1 Gene as a Tumor Suppressor Gene in Hepatocellular Carcinoma Detected by Triple-Combination Array Analysis. Int J Oncol (2013) 43:88–94. 10.3892/ijo.2013.1951 23695389

[42] Ding J Yeh CR Sun Y Lin C Chou J Ou Z. Estrogen Receptor Beta Promotes Renal Cell Carcinoma Progression via Regulating LncRNA HOTAIR-miR-138/200c/204/217 Associated CeRNA Network. Oncogene (2018) 37:5037–53. 10.1038/s41388-018-0175-6 29789714

[43] Karim MA Samad A Adhikari UK Kader MA Kabir MM. A Multi-Omics Analysis of Bone Morphogenetic Protein 5 (BMP5) mRNA Expression and Clinical Prognostic Outcomes in Different Cancers Using Bioinformatics Approaches. Biomedicines (2020) 8(2):19. 10.3390/biomedicines8020019 PMC7168281 31973134

[44] Klonowska K Czubak K Wojciechowska M Handschuh L Zmienko A Figlerowicz M. Oncogenomic Portals for the Visualization and Analysis of Genome-Wide Cancer Data. Oncotarget (2016) 7:176–92. 10.18632/oncotarget.6128 PMC4807991 26484415

[45] Yi JW Kim SJ Kim JK Seong CY Yu HW Chai YJ. Upregulation of the ESR1 Gene and ESR Ratio (ESR1/ESR2) Is Associated With a Worse Prognosis in Papillary Thyroid Carcinoma: The Impact of the Estrogen Receptor Alpha/Beta Expression on Clinical Outcomes in Papillary Thyroid Carcinoma Patients. Ann Surg Oncol (2017) 24:3754–62. 10.1245/s10434-017-5780-z 28124274

[46] Karn T Jiang T Hatzis C Sänger N El-Balat A Rody A. Association Between Genomic Metrics and Immune Infiltration in Triple-Negative Breast Cancer. JAMA Oncol (2017) 3:1707–11. 10.1001/jamaoncol.2017.2140 PMC5824276 28750120

[47] Ferris RL Blumenschein G Jr Fayette J Guigay J Colevas AD Licitra L. Nivolumab for Recurrent Squamous-Cell Carcinoma of the Head and Neck. N Engl J Med (2016) 375:1856–67. 10.1056/NEJMoa1602252 PMC5564292 27718784

[48] Bellmunt J de Wit R Vaughn DJ Fradet Y Lee JL Fong L. Pembrolizumab as Second-Line Therapy for Advanced Urothelial Carcinoma. N Engl J Med (2017) 376:1015–26. 10.1056/NEJMoa1613683 PMC5635424 28212060

[49] Reck M Rodríguez-Abreu D Robinson AG Hui R Csőszi T Fülöp A. Pembrolizumab *Versus* Chemotherapy for PD-L1-Positive Non-Small-Cell Lung Cancer. N Engl J Med (2016) 375:1823–33. 10.1056/NEJMoa1606774 27718847

[50] Zong L Mo S Yu S Zhou Y Zhang M Chen J. Expression of the Immune Checkpoint VISTA in Breast Cancer. Cancer Immunol Immunother (2020) 69:1437–46. 10.1007/s00262-020-02554-3 PMC11027729 32266446

[51] Soliman H Khalil F Antonia S. PD-L1 Expression Is Increased in a Subset of Basal Type Breast Cancer Cells. PloS One (2014) 9:e88557. 10.1371/journal.pone.0088557 24551119 PMC3925108

[52] Liu L Shen Y Zhu X Lv R Li S Zhang Z. ERalpha is a Negative Regulator of PD-L1 Gene Transcription in Breast Cancer. Biochem Biophys Res Commun (2018) 505:157–61. 10.1016/j.bbrc.2018.09.005 30241942

