# Supplementary figures and images for: Pan-Cancer Prognostic Role and Targeting Potential of the Estrogen-Progesterone Axis
Source: Front Oncol. 2021 Jul 12;11:636365. doi: 10.3389/fonc.2021.636365 (PMC8311599; doi:10.3389/fonc.2021.636365)

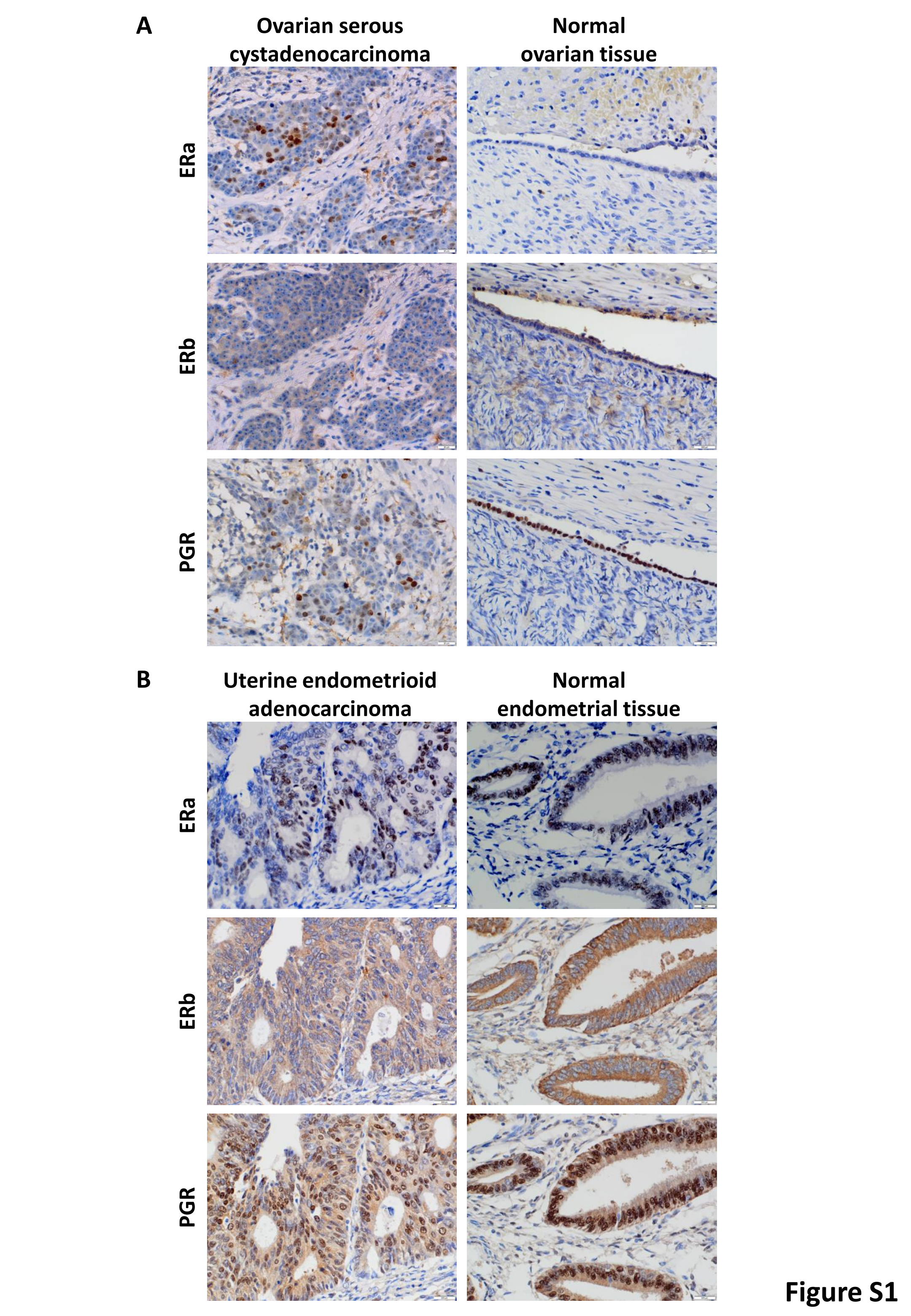

Supplement: Supplementary file 1 [file Image_1.tif]
